# Effect of Mn Content and Heat Treatment on Microstructure and Properties of Laser Cladding of FeCoNiCrTi High-Entropy Alloy Coating

**DOI:** 10.3390/ma18225160

**Published:** 2025-11-13

**Authors:** Shibang Ma, Yicheng Zhou, Congzheng Zhang, Zhengchun Xu, Chengguo Fu

**Affiliations:** 1 School of Intelligent Manufacturing and Electrical Engineering, Nanyang Normal University, Nanyang 473061, China; mshibang@nynu.edu.cn (S.M.); zhouzyc@nynu.edu.cn (Y.Z.); zcz1010@126.com (C.Z.); zcxnynu@163.com (Z.X.); 2 Collaborative Innovation Center of Intelligent Explosion-Proof Equipment, Henan Province, Nanyang 473061, China; 3 School of Mechanical Engineering, Guizhou University of Engineering Science, Bijie 551700, China

**Keywords:** laser cladding, FeCoNiCrTiMnx coating, heat treatment, microstructure evolution, microhardness, friction coefficient

## Abstract

In this study, the effects of different Mn content and heat treatment on the microstructure and properties of CoCrFeNiTi coatings by laser cladding technology were investigated. Scanning electron microscopy, energy-dispersive spectrometry, and X-ray diffraction were used to analyze the structure and composition. The hardness and wear resistance were tested by a microhardness tester and a friction-wear tester. The results show that there are many intermetallic compounds rich in Ti and Ni between the grains. As the Mn content increases, the coating gradually transitions from a dual-phase structure of BCC and FCC to a single FCC structure. The hardness of the coating decreases gradually with the increase in Mn content due to the change in the phase structure, while the friction coefficient decreases slightly at first and then increases significantly. The main wear mechanisms of the coating are adhesive wear and abrasive wear. After heat treatment at 600 °C, petal-like Laves precipitates appear. The average microhardness of CoCrFeNiTi coatings after heat treatment is lower than before treatment, and the friction coefficient is higher than before treatment. The average microhardness of the coating increases slightly with the increase in the treatment temperature. The average friction coefficient of the coating obtained after heat treatment at 600 °C is only 0.5941 because of its uniform microstructure. Therefore, it is reduced by approximately 15% compared with the base metal.

## 1. Introduction

Since Ye and others [1] proposed the design concept of high-entropy alloys (HEAs), these new alloys have become a research hotspot in the field of materials [2,3,4]. Due to their outstanding properties, such as excellent wear resistance [5,6], corrosion resistance [7,8], mechanical properties [9,10,11], and electrical conductivity [12], HEAs are considered an advanced material with promising applications in many industrial fields.

Most HEAs of the CoCrFeNi series have a simple solid solution structure, and this series of alloys has excellent performance, including high-temperature properties, ductility, creep resistance, and corrosion resistance [13,14,15]. Ye et al. [16] prepared a CrMnFeCoNi high-entropy alloy coating on the surface of A36 steel by adding the Mn element. No pores or cracks were found in the coating, and the coating had good metallurgical bonding with the matrix. Yuan et al. [17] introduced 0.2 at. % Ti into CoCrFeNi by vacuum arc melting. CoCrFeNiTi0.2 achieved an excellent combination of strength and plasticity. The yield strength at 77 K was 1.0 GPa, the ultimate tensile strength was 1.5 GPa, and the ductility was 35%. The multi-scale precipitates interacted with the dislocation in different ways, resulting in synergistic effects that enhanced the strengthening and toughening properties. Zhao [18] prepared a CoCrFeNiTi coating by laser powder bed melting technology using 0.6 at. % Ti. The addition of Ti promoted the formation of the second phase, which increased the microhardness to about 600 HV. The second phase of strengthening contributions dominated. In related research work, Liu et al. [19] further increased the content of the Ti element and prepared a CoCrFeNiTi coating by the laser cladding method. The increase in microhardness (700 HV) of the coating was mainly due to the strengthening of the second phase. Chen et al. [20] prepared a Ti-modified CoCrFeNiMnTi_x_ high-entropy alloy by vacuum arc melting. The Ti-rich body-centered cubic phase was observed in CoCrFeNiMnTi0.25 and CoCrFeNiMnTi0.55. The number of titanium-rich phases increased with the increase in titanium content. At the same time, the mechanical properties of CoCrFeNiMnTi_x_ were significantly improved. When the Ti content was 0, 0.25, and 0.55, the microhardness was 175 HV, 253 HV, and 646 HV, respectively, and there was an obvious increasing trend, while the plasticity decreased. In spite of this, research on the preparation of CoCrFeNiTiMn high-entropy alloy by adding Ti and Mn at the same time is still relatively scarce.

In this study, CoCrFeNiTiMnx high-entropy alloy coating was prepared by laser cladding technology [7,21], and the related effects of different Mn content on the microstructure and properties of CoCrFeNiTi coating were investigated. At the same time, heat treatment was carried out on the CoCrFeNiTi laser cladding coating to study its structural and performance changes.

## 2. Materials and Methods

**Figure 1:**
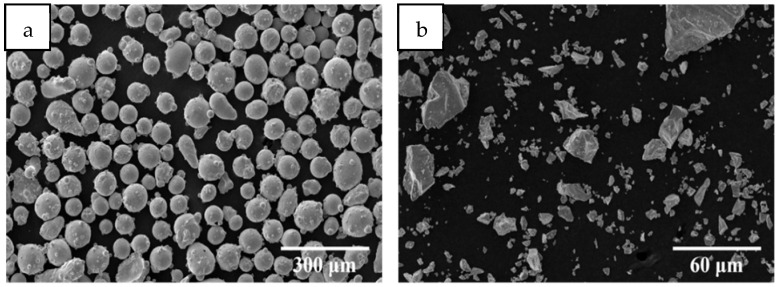
Micromorphology of HEA and Mn powder: (**a**) HEA; (**b**) Mn.

The base material of the cladding experiment was a 45# steel plate with a size of 120 mm × 120 mm × 10 mm. The basic composition is shown in Table 1. The surface of the sample was sanded smooth with sandpaper, washed with anhydrous ethanol, dried by blowing, and then stored in a drying oven. The metal powder used for cladding was CoCrFeNiTi high-entropy alloy powder with an equal atomic ratio and Mn powder with a purity of 99.8%. The SEM micrographs are shown in Figure 1. The elements of CoCrFeNiTiMnx (x = 0.0, 0.2, 0.4, 0.6, 0.8) are shown in Table 2. When preparing the required high-entropy alloy powder, it was weighed with precision electronics (accuracy is ±0.1 mg). Then, the weighed powder was ground and mixed in an agate mortar for 2 h, and the obtained powder was loaded into the sample tube for use. Samples with different Mn content are denoted as Mn0, Mn02, Mn04, Mn06, and Mn08, respectively.

The laser cladding was carried out by the RFL-C4000 fiber laser robot system (Raycus Fiber Laser Technologies Co., Ltd., Wuhan, China). In order to achieve a cladding layer with good quality, no obvious cracks, and relatively large melting width and build-up height, the parameters obtained from preliminary experiments were as follows: laser power of 2.2 kW, laser spot diameter of 3 mm, laser scanning speed of 5 mm/s, and overlap ratio of 50%. The wavelength of the laser was 1070 ± 10 nm, and the focus distance was 2–3 cm from the sample. Pure argon gas was used as the shielding gas, with a flow rate of 15 L/min. The coating thickness after laser cladding was about 1–1.5 mm. An XT-1200 tubular resistance furnace (Xinyu Furnaces Co., Ltd., Nanyang, China) was used to heat the CoCrFeNiTi coating at 400 °C, 600 °C, and 800 °C, respectively. After heating to the specified temperature, the coating was kept for 30 min and finally cooled in the air. Samples with different heat treatment temperatures are denoted as HT400, HT600, and HT800.

To investigate the microstructure and chemical composition of the samples, some 10 mm × 10 mm × 10 mm specimens were cut by wire cut electrical discharge machining (WEDM). A mixture of 5% HNO_3_ and 17% HF was used to corrode the cross section of the sample after grinding, polishing, and cleaning to observe the microstructure. The phase composition of the cladding coating was analyzed by X-ray diffraction (XRD, Japanese physical SmartLab, Tokyo, Japan) with a diffraction range of 10–80° and a scanning speed of 8°/min. A scanning electron microscope (SEM, SEM3200C, CIQTEK Co., Ltd., Hefei, China) with energy-dispersive (EDS, Aztec Energy ES, X-Max 20, Oxford, UK) function was used to observe the coating. An HXS-1000AY microhardness tester (HIGHWELL Optoelectronics Technology Co., Ltd., Shanghai, China) was used to test the hardness of the coating. The test was evenly spaced from top to bottom. The load was 0.2 kg, and the holding time was 15 s. The GF-1 friction and wear testing machine was used to test the coated surface of multiple samples, and the change curve of the friction coefficient was obtained. During the experiment, GCr15 steel balls with a diameter of 5 mm were used for testing. The test parameters were as follows: test time of 30 min, load of 8 N, motor speed of 300 rpm, and reciprocating length of 5 mm. After the test, SEM was used to observe the wear surface and analyze the wear mechanism.

## 3. Results and Discussion

### 3.1. Effect of Mn Content on Coatings

#### 3.1.1. Microstructure Characterization

**Figure 2:**
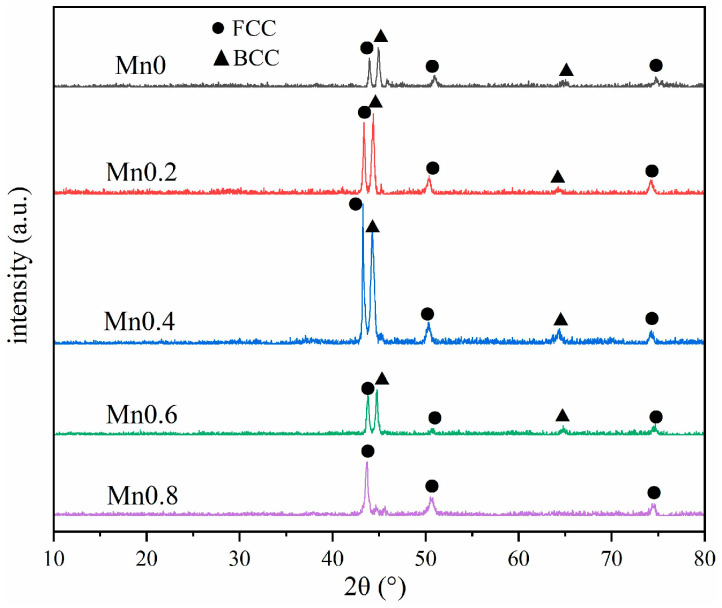
XRD pattern of different Mn content coatings.

The XRD diffraction pattern of the coating is shown in Figure 2. It can be observed that the coating without Mn and Mn02 is mainly a biphase structure composed of FCC and BCC due to the presence of Ti. At x = 0.4, the diffraction intensity of the FCC phase exceeds that of the BCC phase. Mn08 coating has almost no BCC structure, with only a single FCC phase. The transformation of the coating structure is mainly due to the fact that Mn is an austenite-forming element, and the addition of Mn will promote the formation of the FCC phase and reduce the number of BCC phases.

**Figure 3:**
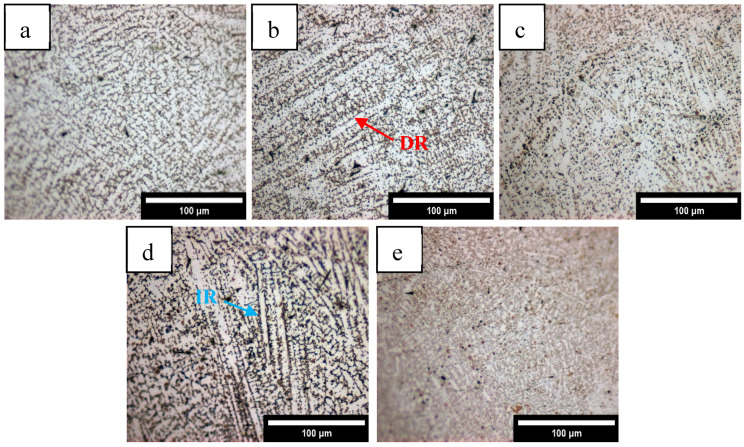
Metallographic images of the coatings with different Mn content: (**a**) Mn0, (**b**) Mn02, (**c**) Mn04, (**d**) Mn06, (**e**) Mn08.

After the surface of the coating was polished and etched, the metallography observed by an optical microscope is shown in Figure 3. The five coatings with different Mn content are composed of dendrites and equiaxed crystals. With the increase in Mn content, the proportion of dendrites (Figure 3b) increases, and the grains become finer. It can be observed that most of the coatings are dendritic dendrites growing along the thermal conductivity direction of the molten pool [21]. As the solidification boundary moves towards the middle of the molten pool, the cooling rate (R) gradually increases, and the temperature gradient (G) decreases, resulting in subcooling of the components, and the grains gradually grow in the form of dendrites [23]. During crystallization of the dendrite crystal axis, latent heat is released to both sides of the liquid phase, resulting in a negative temperature gradient in the direction perpendicular to the crystal axis, resulting in a secondary crystal axis on the crystal axis [24]. At the same time, it can be seen that the grain orientation is directional in a small range but is relatively chaotic on the whole. This is mainly because the laser energy density is high in the cladding process, and the laser is constantly moving forward, resulting in an uneven distribution of the temperature gradient in the molten pool [25]. Where the temperature gradient is non-uniform across the solid–liquid interface, the growth direction of dendrites becomes inconsistent and appears irregular.

**Figure 4:**
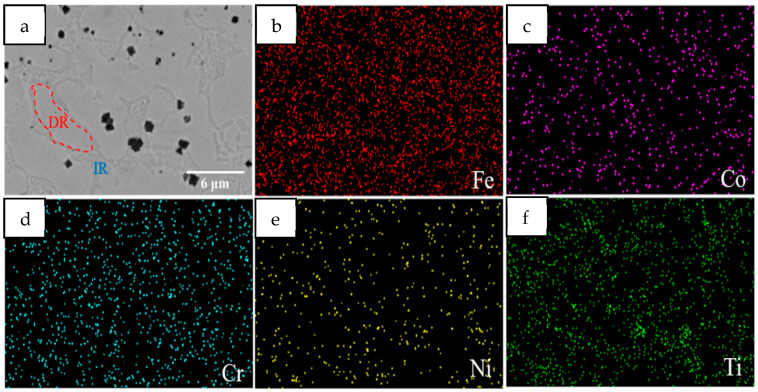
SEM micrographs of the Mn0 coating: (**a**) backscattered electron images, (**b**–**f**) EDS area scanning element distribution.

Figure 4 shows the SEM images of the Mn0 coating, which has a uniform contrast in the backscatter electron images. Figure 4a shows clear dendrites (secondary dendrites (DRs)) and interdendrite regions (IRs), and a small amount of lamellar structures are distributed in the IR. Combined with the element surface distribution mapping of Figure 4b–f, it mainly contains two elements, Ti and Ni, so it may include the η phase (Ni_3_Ti), R phase (Ni_2_Ti), δ phase (NiTi_2_), and other intermetallic compounds [26,27,28]. At the same time, there are black block precipitates in the coating, mainly distributed near the IR, which will be analyzed in conjunction with EDS.

**Figure 5:**
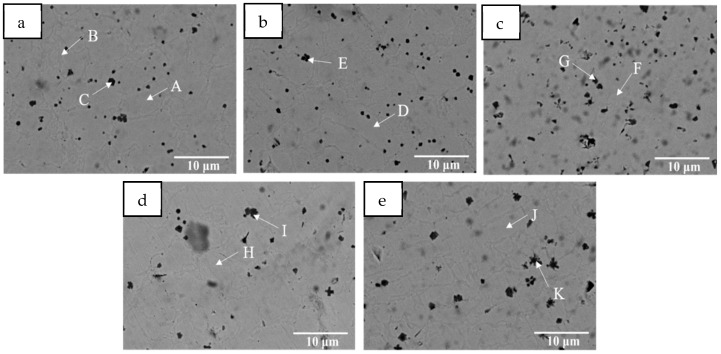
SEM images of the coatings with different Mn content: (**a**) Mn0, (**b**) Mn02, (**c**) Mn04, (**d**) Mn06, (**e**) Mn08.

Figure 5 shows SEM micrographs of five coatings. As can be seen, the addition of Mn does not alter the dendritic morphology of the coatings. However, with the increase in Mn content, the number of black precipitates in the coating shows a phenomenon of first increasing and then decreasing. EDS elemental scanning was performed on the internal punctuation points of the coating, as shown in Figure 5, and the results are shown in Table 3. The HEA matrix represented by point A in Figure 5a shows that the content of Cr and Co components is basically the same, while the content of Ni and Ti in the IR (point B) is significantly higher than that in the intragranular region (point A). This is consistent with the mapping results in Figure 4, indicating that the IR contains more Ni- and Ti-rich intermetallic compounds. Due to the high dilution rate caused by the high input of laser cladding, Fe in the base metal enters the coating, resulting in a significant increase in its content. Point C represents dark massive tissue, mainly complex carbides of Ti and Fe. The presence of C may be due to two reasons. The first is that the C element within the substrate enters the coating during the cladding process, causing a certain dilution effect. The second is that the residual C element from the glue used during the pre-powder coating enters the coating. Analysis of the punctuation points in Figure 5b–e reveals that as the amount of Mn added increases, the amount of Mn in the coating also increases, and the content of Ti in the carbides also significantly increases. This may be due to Mn replacing the original Ti within the unit cell, resulting in more Ti migrating and binding with C. At the same time, small-sized carbides undergo Ostwald ripening, resulting in an overall decrease in the number of carbides, an increase in size, and an increase in Ti content.

#### 3.1.2. Microhardness

**Figure 6:**
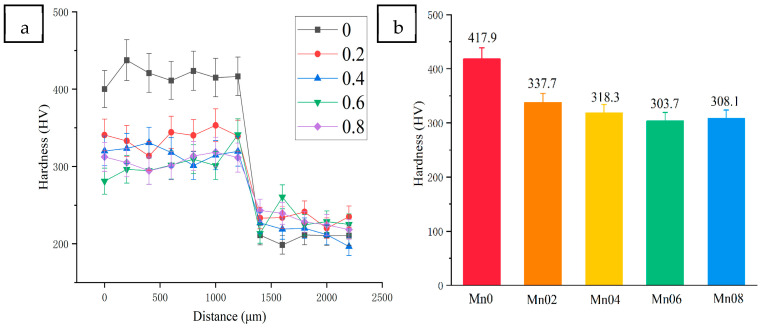
Microhardness of coatings with different Mn content: (**a**) the curve of the microhardness test, (**b**) comparison of average hardness.

Figure 6 shows the contrast curve of microhardness and average hardness of coatings with different Mn content, with the dashed line representing the interface of the coatings and the base metal. The average microhardness of the Mn0 coating is the highest, reaching 415.4 HV, and the hardness gradually decreases with the increase in Mn content. The higher microhardness is due to the strengthening of the solid solution caused by Ti atoms with larger atomic radii, which leads to lattice distortion. Huang et al. [29] also reported that a large difference between the atomic radii of alloying elements would produce a solid solution strengthening effect, which would also lead to lattice distortion and large residual compressive stress in the coating. The reduction in microhardness is related to the transition from FCC+BCC+intermetallic compounds (Ni_3_Ti, Ni_2_Ti, and NiTi_2_) to FCC+BCC biphase and then to FCC single phase. This is because the BCC phase has higher slip resistance [30], and the intermetallic compounds are harder than the FCC and BCC phases, resulting in a second-phase strengthening effect. The hardness of the base metal is small, and the average hardness is about 225 HV, which is significantly lower than that of all HEA coatings (about 83.1 HV~192.9 HV).

#### 3.1.3. Tribological Properties

**Figure 7:**
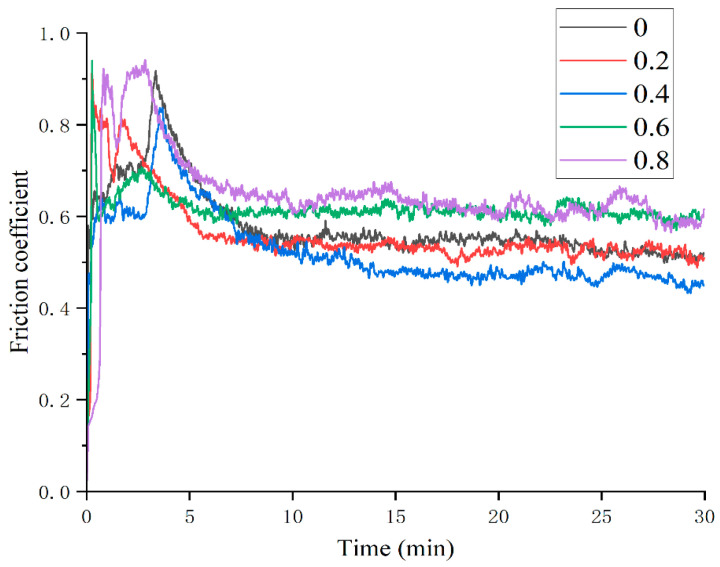
Friction curves of coatings with different Mn content.

Figure 7 shows the variation in coating friction coefficient with different Mn content. It can be seen that the friction and wear process of the five groups of samples all go through the stage of severe wear to stable wear, which may be due to the fact that the surface roughness of the GCr15 steel ball is relatively large when it comes into contact with the coating surface and gradually becomes stable with the wear. The average friction coefficient of the five groups of coatings calculated by the friction curve is shown in Table 4. There is little difference between the average friction coefficient of Mn0, Mn02, and Mn04 coatings, among which Mn04 is the smallest at 0.5236. When Mn content is higher, the average friction coefficient of Mn06 and Mn08 coatings increases significantly, reaching 0.6168 and 0.6480, respectively.

**Figure 8:**
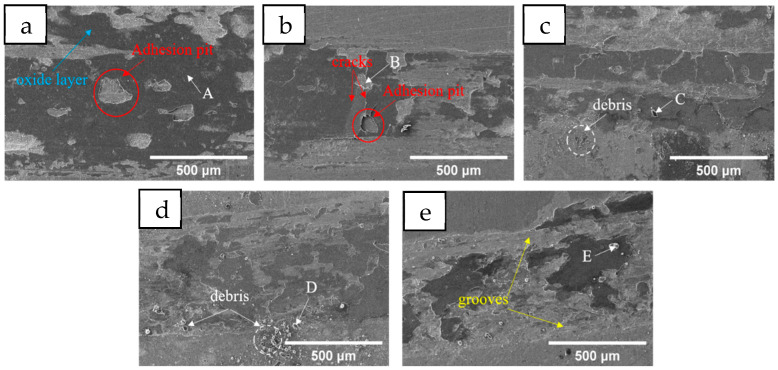
The worn surface morphology of the coatings with different Mn content: (**a**) Mn0, (**b**) Mn02, (**c**) Mn04, (**d**) Mn06, (**e**) Mn08.

The change trend of the friction coefficient is not completely consistent with the change in coating hardness, so the analysis is made in combination with the wear morphology of each coating shown in Figure 8 and the EDS testing in Table 5. A dark oxide layer appears on the surface of all five groups of coatings (point A). After the coatings of Mn0 and Mn02 are worn, obvious sticking pits appear on the surface of the oxide layer (red circles in Figure 8a,b). From Figure 8b, it can be observed that the oxide layer at the adhesive pit, when damaged, causes crack propagation, lifting the nearby oxide layer. In the next wear, it ruptures and peels off, exposing the underlying coating and initiating further oxidation (point B), thus repeating the cycle. Adhesive wear is a phenomenon caused by the relative movement of solids; when the contact of two surfaces has relative movement, the adhesive point is destroyed, and the material is transferred from the surface of one part to the surface of another part [31]. When Mn content reaches 0.4, fine debris (white circle in Figure 8c) begins to appear on the wear surface (point C). Due to the relatively low oxygen content and hardness, the exuded oxide layer will soon be ground into small particles. When the size of the abrasive particles is small and the hardness is relatively low, this debris will play a certain lubricating role [32]. This is why the mean friction coefficient of the Mn04 coating is the lowest, but it is not significantly lower than that of the Mn0 and Mn02 coatings. However, a large amount of large-sized debris (points D and E) appears in the Mn06 and Mn08 coatings, with significantly increased O content and relatively high hardness. This may be due to Mn being a reactive element that is prone to oxidation. During the friction process, although the overall temperature increase is very small, within a local range, due to the rapid friction between the grinding material and the coating, the instantaneous temperature can reach a very high level, thus promoting oxidation. There have been two hypotheses about oxidative wear for a long time: (1) most oxidation occurs at the moment the original metal is exposed, and subsequent further contact only leads to the shearing of the oxide at the oxide–metal interface; (2) an equal amount of oxidation occurs at each contact until the critical oxidation thickness is reached, and beyond this thickness, shear occurs at the oxide–metal interface [33]. The second theory was later proved to be more in line with reality. Large-sized, high-hardness debris causes abrasive wear at the same time as adhesive wear. Even the furrow caused by the plastic deformation of the abrasive particles into the coating has been observed in the Mn08 coating [34], which is the main reason for the significant increase in the average friction coefficient of the Mn06 and Mn08 coatings.

### 3.2. Effect of Heat Treatment on Coatings

#### 3.2.1. Microstructure Characterization

**Figure 9:**
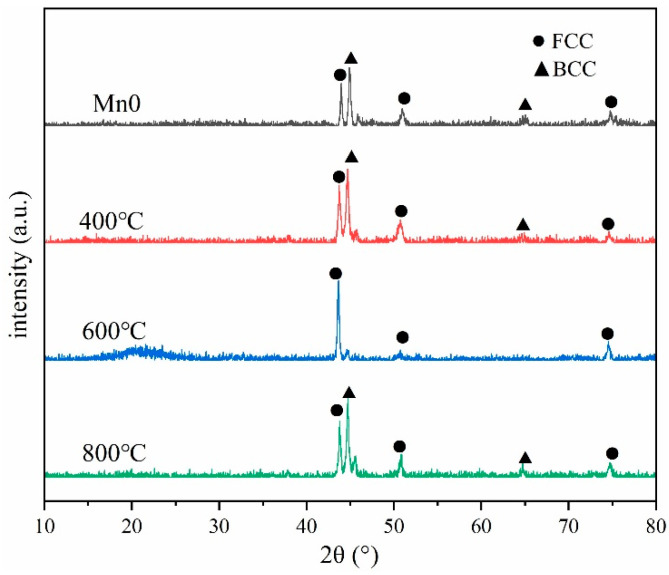
XRD pattern of different HT temperatures.

Generally speaking, the heating and cooling speed of laser cladding is fast, so there will be internal stress or internal defects such as pores and cracks in the cladding coating [35]. Therefore, researchers often use heat treatment processes to regulate the structure and properties of the coating [36,37]. The XRD diffraction results of the coating after heat treatment are shown in Figure 9. The analysis shows that the phase of the coating after heat treatment at 400 °C and 800 °C is similar to that of the Mn0 coating, both of which are FCC and BCC biphase structures, while after heat treatment at 600 °C, the BCC phase of the coating almost disappears, leaving only a single FCC phase structure. At the same time, the diffraction strength of the coating after heat treatment is improved, which indicates that the heat treatment reduces the internal defects of the coating and improves the crystallinity of the coating.

**Figure 10:**
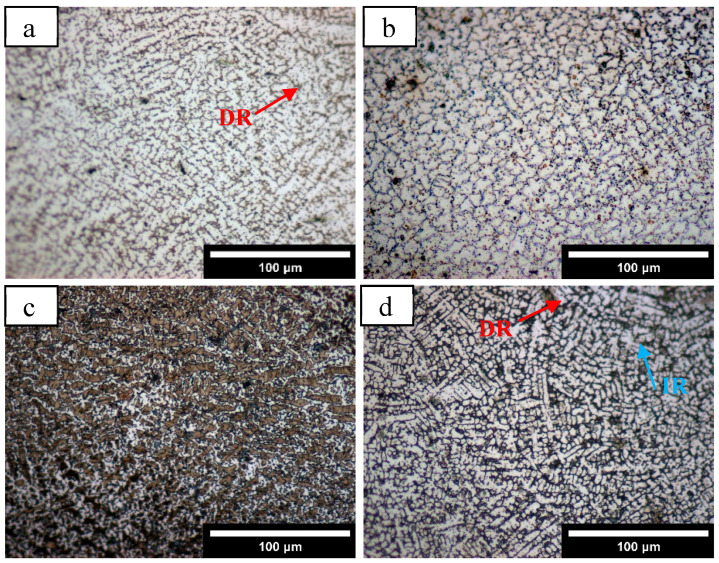
Metallographic images of the coatings with different HT temperatures: (**a**) Mn0, (**b**) 400 °C, (**c**) 600 °C, (**d**) 800 °C.

**Figure 11:**
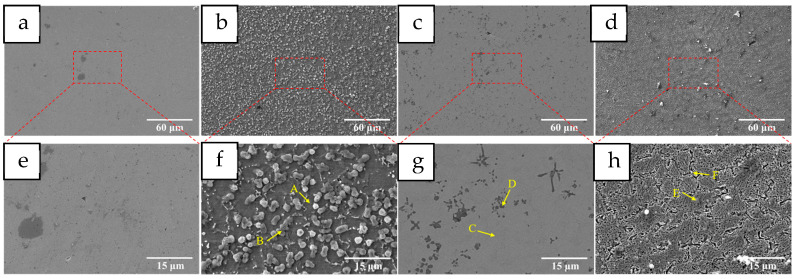
SEM images of the coatings with different HT temperatures: (**a**) Mn0, (**b**) 400 °C, (**c**) 600 °C, (**d**) 800 °C, (**e**–**h**) partial enlarged detail.

Figure 10 shows the metallographic structure of the coatings at different heat treatment temperatures. After heat treatment at 400 °C, the grain size of the coating becomes larger, and the dendrite structure is not obvious. At the same time, small dark precipitation appears in the grain boundary region. This shows that heat treatment can reduce the internal stress of the coating, release the distortion, and promote the diffusion of elements and the migration of grain boundaries. When the heat treatment temperature increases to 600 °C, the grain boundaries of the coating are not obvious, some grain boundaries break, and the grains begin to fuse. After heat treatment at 800 °C, the coating returns to a dendrite structure, the grain size decreases, the amount of dark precipitation in the grain boundary region increases, and the size increases. Figure 11 shows the SEM microscopic image of the coating before and after heat treatment, in which the coating of HT400 shows a uniform distribution of many micron-scale massive tissues, whose size is about 2.5 μm when observed at magnification. These massive tissues are not inside the coating but attached to the surface of the coating. In the HT600 coating, it can be observed that the HEA matrix maintains a uniform contrast, the coating structure is relatively dense, and there are blocky and petal-like precipitation phases in the coating. On the other hand, the obvious dendrite structure can be observed in the HT800 coating. At the same time, the inner grain is no longer dense, showing a lamellar structure; the grain boundary width increases, and bright fine precipitates exist at the edge of the grain boundary. At the same time, a small amount of adhesion exists on the surface of the coating.

EDS element tests were carried out for each punctuation area in the SEM image, and the results are shown in Table 6. It can be seen that the attachments on the surface of the HT400 coating are mainly carbides and oxides of each element. Since the sample is cooled in the air after the heat treatment and insulation, the attachments will adhere to the surface of the coating. It is worth noting that in addition to C, there is a dark precipitation in the HT600 coating. The combined content of the four elements Cr, Ni, Co, and Fe is about twice that of Ti, which indicates that this precipitation may be the Laves phase of Co_2_Ti [38,39], where Cr, Ni, and Fe replace some Co atoms. However, there are oxides and carbides in the grain boundary region of the HT800 coating, and the Ti content in the grain boundary region is significantly higher than that in the grain. These results show that the heat treatment process significantly promotes the diffusion of elements, especially Ti, which has a strong bond with other elements. Various precipitated phases are formed by the release of internal stress of the coating during the heat treatment process, and the extremely high content of Fe also leads to the generation of oxides.

#### 3.2.2. Microhardness

**Figure 12:**
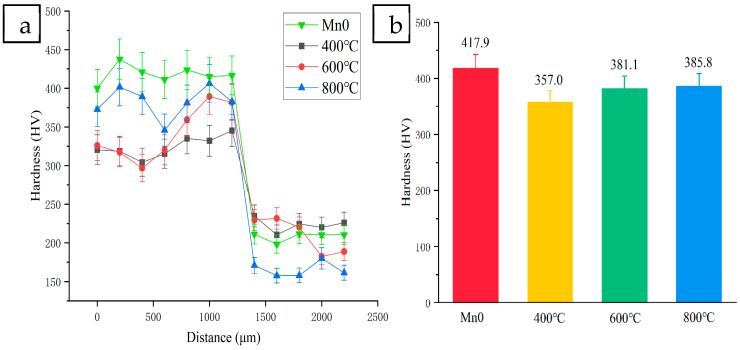
Microhardness of coatings with different HT temperatures: (**a**) the curve of the microhardness test, (**b**) comparison of average hardness.

The microhardness changes after heat treatment are shown in Figure 12. Compared with before heat treatment, the microhardness of the coating after heat treatment decreases. The release of the distortion energy of the coating results in the internal dislocation density decreasing and the coating hardness decreasing. With the increase in heat treatment temperature, the hardness of the coating increases slightly, and the average microhardness reaches 385.8 HV at 800 °C. This is because with the increase in heat treatment temperature, the diffusion rate of elements in the coating increases, and more Laves phase, carbide, and oxide precipitation appear, which play a strengthening role.

#### 3.2.3. Tribological Properties

**Figure 13:**
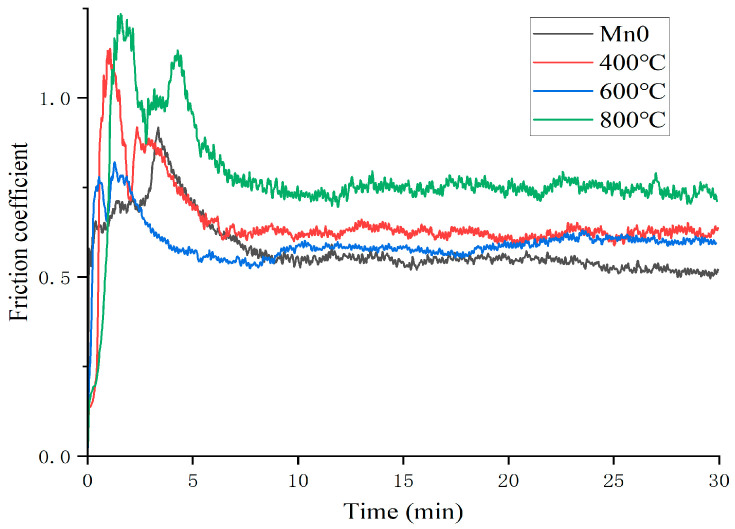
Friction curves of coatings with different HT temperatures.

**Figure 14:**
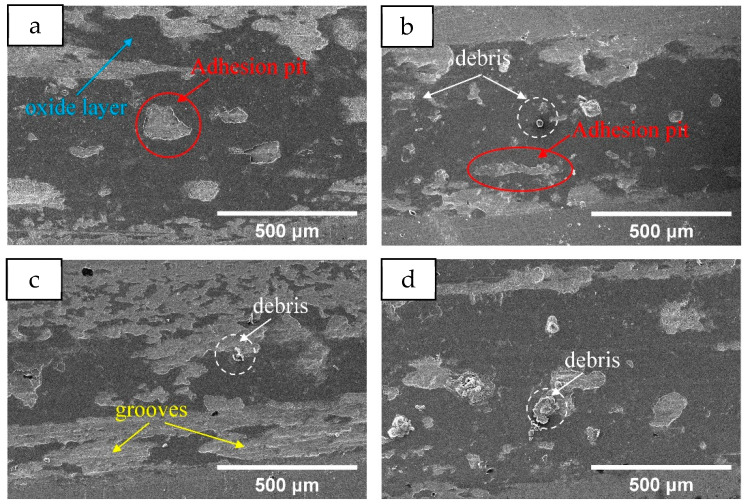
The worn surface morphology of the coatings with different HT temperatures: (**a**) Mn0 (**b**) 400 °C (**c**) 600 °C (**d**) 800 °C.

Figure 13 shows the change curve of the friction coefficient of the Mn0 coating after heat treatment. As can be seen from the figure, the friction coefficient of the coating fluctuates greatly after heat treatment; the friction coefficient at 400 °C and 800 °C even exceeds 1 at the beginning, which indicates that the grinding ball and the coating appear stuck at the initial wear. The average friction coefficient obtained by analysis is shown in Table 7. The average friction coefficient of the HT600 coating is the smallest at 0.5941, while that of the HT800 coating is 0.7781. After heat treatment, the average friction coefficient increases due to the decrease in the hardness of the coating. According to the observation of wear morphology in Figure 14, the wear mechanism of the Mn0 coating is adhesive wear. After heat treatment, there is some debris in the wear morphology. The HT600 coating has the least debris, its oxidation layer area is small, the oxidation degree is relatively low during the wear process, and there are obvious furrows, indicating that its uniform structure is conducive to improving its friction resistance. The large debris present in the HT800 coating will become new abrasive particles, resulting in a significant increase in its friction coefficient.

## 4. Conclusions

In this paper, CoCrFeNiTiMnx high-entropy alloy coating was prepared on 45# steel by laser cladding, and the effect of Mn content on the microstructure and properties of the coating was studied. The CoCrFeNiTi coating was further heat-treated at 400 °C, 600 °C, and 800 °C to explore the effects of different treatment temperatures on the microstructure and properties of the coating. The main conclusions are summarized as follows:

(1) HEA coatings with different Mn content prepared by laser cladding have a typical dendrite structure. With the increase in Mn content, the microstructure gradually changes from FCC+BCC dual phase to a single FCC structure.
(2) The average microhardness of Mn0 coating is the highest, reaching 415.4 HV. With the increase in Mn content, the coating hardness has a gradual decline, which is related to the transition from FCC+BCC+intermetallic to FCC+BCC and then to FCC single phase. The change trend of the friction coefficient is not completely consistent with that of the coating hardness. In addition to adhesive wear, abrasive wear also occurs, which is the main reason for the significant increase in the friction coefficient of the Mn06 and Mn08 coatings.
(3) After heat treatment at 400 °C and 800 °C, the Mn0 coating exhibits a dual-phase FCC+BCC structure, whereas a single FCC structure is retained following treatment at 600 °C. The HT400 coating surface is primarily decorated with carbides and oxides of various elements, approximately 2.5 μm in size. The HT600 coating maintains a uniform contrast and contains blocky and petal-like Co_2_Ti-type Laves phases. In the HT800 coating, Ti-rich oxides and carbides are present along the grain boundaries.
(4) Compared with the Mn0 coating, the microhardness of the heat-treated coatings decreases, which is attributed to the release of distortion energy and a corresponding reduction in dislocation density. As the heat treatment temperature increases, more Laves phases, carbides, and oxide precipitates form, leading to a slight increase in hardness. The average friction coefficient increases after heat treatment due to the reduced coating hardness.

## Figures and Tables

**Table 1:** The chemical composition of 45# steel substrate (wt. %) [22].

C	Si	Mn	P	S	Cr	Ni	Fe
0.42–0.5	0.17–0.37	0.50–0.80	≤0.035	≤0.035	≤0.25	≤0.25	Bal.

**Table 2:** The chemical composition of HEA coatings (at. %).

	Co	Cr	Fe	Ni	Ti	Mn
CoCrFeNiTi	20.00	20.00	20.00	20.00	20.00	0.00
CoCrFeNiTiMn0.2	19.23	19.23	19.23	19.23	19.23	3.85
CoCrFeNiTiMn0.4	18.52	18.52	18.52	18.52	18.52	7.41
CoCrFeNiTiMn0.6	17.86	17.86	17.86	17.86	17.86	10.71
CoCrFeNiTiMn0.8	17.24	17.24	17.24	17.24	17.24	13.79

**Table 3:** EDS element test results at each point in Figure 5 (at. %).

	Elements	Cr	Ni	Co	Ti	Fe	Mn	C
HEA coating	A	12.48	9.00	10.51	5.86	62.15	—	—
	B	8.01	13.84	12.03	15.06	51.05	—	—
	D	10.91	11.33	11.68	8.67	55.20	2.22	—
	F	7.68	9.80	9.19	5.86	61.95	5.53	—
	H	8.71	11.04	12.17	15.07	46.73	6.27	—
	J	9.02	15.48	13.34	12.67	41.92	7.57	—
carbides	C	3.92	3.33	3.67	14.33	17.52	—	57.22
	E	2.31	2.76	2.53	16.47	11.13	0.47	64.33
	G	1.63	1.49	1.37	22.50	17.59	0.76	54.67
	I	2.80	1.80	2.42	32.42	12.40	1.28	46.88
	K	1.58	2.81	2.37	31.72	13.98	1.74	45.79

**Table 4:** Average friction coefficient of coatings with different Mn content.

Samples	Mn0	Mn02	Mn04	Mn06	Mn08
Friction coefficient	0.5794	0.5604	0.5236	0.6168	0.6480

**Table 5:** EDS element test results at each point in Figure 8 (at. %).

Elements	Cr	Ni	Co	Ti	Fe	Mn	O
A	3.24	2.51	2.68	2.94	30.99	—	57.64
B	8.23	9.32	9.06	6.46	59.16	1.95	5.82
C	1.78	1.38	—	1.10	63.25	0.78	31.70
D	1.88	1.47	—	1.32	51.41	0.96	42.95
E	1.20	—	—	0.92	54.50	1.04	42.34

**Table 6:** EDS element test results at each point in Figure 11 (at. %).

Elements	Cr	Ni	Co	Ti	Fe	C	O
A	5.26	2.29	5.22	1.35	85.88	—	—
B	2.42	2.03	3.09	0.65	50.43	32.40	8.97
C	10.70	10.97	12.00	3.58	62.75	—	—
D	2.87	4.67	4.80	16.56	12.59	58.50	—
E	6.18	5.24	5.11	2.14	46.83	34.49	—
F	7.16	8.34	7.52	7.81	42.96	20.38	5.83

**Table 7:** Average friction coefficient of coatings with different HT temperatures.

	Mn0	HT400	HT600	HT800
Friction coefficient	0.5794	0.6524	0.5941	0.7781

## Data Availability

The original contributions presented in the study are included in the article. Further inquiries can be directed to the corresponding author.
